# Transcriptional Profiling Reveals Lineage-Specific Characteristics in ATR/CHK1 Inhibitor-Resistant Endometrial Cancer

**DOI:** 10.3390/biom16010169

**Published:** 2026-01-20

**Authors:** Tzu-Ting Huang, Jung-Min Lee

**Affiliations:** Women’s Malignancies Branch, Center for Cancer Research, National Cancer Institute, National Institutes of Health, Bethesda, MD 20892, USA; leej6@mail.nih.gov

**Keywords:** endometrial cancer, ATR, CHK1, drug resistance

## Abstract

Recurrent endometrial cancer (EC) has limited therapeutic options beyond platinum-based chemotherapy, highlighting the need to identify exploitable molecular vulnerabilities. Tumors with high genomic instability, including microsatellite instability-high (MSI-h) or copy-number-high (CNH) ECs, rely on the ATR-CHK1 signaling pathway to tolerate replication stress and maintain genome integrity, making this pathway an attractive therapeutic target. However, acquired resistance to ATR and CHK1 inhibitors (ATRi/CHK1i) often develops, and the transcriptomic basis of this resistance in EC remains unknown. Here, we established isogenic ATRi- and CHK1i-resistant cell line models from MSI-h (HEC1A) and CNH (ARK2) EC lineages and performed baseline transcriptomic profiling to characterize stable resistance-associated states. MSI-h-derived resistant clones adopted a unified transcriptional state enriched for epithelial-mesenchymal transition, cytokine signaling, and interferon responses, while ATRi-resistant models showing additional enrichment of developmental and KRAS/Notch-associated pathways. In contrast, CNH-derived resistant clones diverged by inhibitor class, with ATRi resistance preferentially enriching proliferation-associated pathways and CHK1i resistance inducing interferon signaling. Notably, *THBS1*, *EDN1*, and *TENM2* were consistently upregulated across all resistant models relative to parental lines. Together, these findings demonstrate that acquired resistance to ATRi and CHK1i in EC is shaped by both lineage and inhibitor class and provide a transcriptomic framework that may inform future biomarker development and therapeutic strategies.

## 1. Introduction

Endometrial cancer (EC) is the most common gynecologic malignancy in developed countries, with rising global incidence and mortality [1,2]. While early-stage EC is often curable with surgery, advanced or recurrent disease has poor outcomes, and five-year survival remains below 20% [1]. Platinum-based chemotherapy remains the cornerstone of treatment, but responses are often limited and rarely durable [3,4]. Targeted therapies such as immune checkpoint blockade for mismatch repair-deficient tumors and HER2-targeted agents for HER2-positive disease have improved outcomes in selected EC subgroups [3,5,6]. However, these treatments apply only to biomarker-defined populations and are often hindered by acquired resistance [3,5,6]. These challenges highlight the need for strategies that target shared biological vulnerabilities in high-risk EC.

Among the molecular subtypes defined by The Cancer Genome Atlas (TCGA), microsatellite instability-high (MSI-h) and copy-number-high (CNH) tumors account for a substantial proportion of EC-related deaths [7,8,9]. Despite their distinct genomic features, with mismatch-repair deficiency and inflammatory signaling characterizing the MSI-h subtype [9] and chromosomal instability defining *TP53*-mutant CNH tumors [10,11,12], both subtypes exhibit high levels of genomic instability and depend on the ATR-CHK1 pathway to maintain genome integrity and survival [13,14,15]. Preclinical studies demonstrate that inhibition of ATR or CHK1 sensitizes EC cells to DNA-damaging agents and promotes mitotic failure, providing a rationale for the clinical evaluation of agents such as the ATR inhibitor (ATRi) camonsertib and the CHK1 inhibitor (CHK1i) prexasertib [16,17,18,19,20].

Despite the therapeutic promise of ATR and CHK1 inhibition, responses are variable and often not durable. Existing mechanistic studies in other cancer types have primarily examined determinants of intrinsic sensitivity rather than acquired resistance, and most have been conducted outside the EC context. For example, CRISPR-based screens in non-EC models have identified genes such as *CDC25A* [21] and components of the nonsense-mediated decay pathway [22] as modulators of ATRi response in embryonic stem cells or gastric cancer models. In *BRCA*–wild-type ovarian cancer, reduced CDK1/cyclin B1 activity has been associated with prexasertib resistance [23], while epidermal growth factor receptor pathway has been linked to lower baseline sensitivity to prexasertib in triple-negative breast cancer [24]. However, these studies describe acute or engineered resistance rather than stable resistance that emerges after prolonged drug exposure, and they do not incorporate lineage-specific transcriptional characteristics of MSI-h and CNH EC.

To address these questions, we generated isogenic models of acquired resistance to ATRi camonsertib and CHK1i prexasertib in representative MSI-h and CNH EC cell lines and performed transcriptomic analysis to characterize the adaptive states that emerge after chronic checkpoint kinase inhibition. By comparing resistance patterns across lineages and inhibitor classes, our study provides a systematic framework for understanding the transcriptional features associated with acquired ATRi and CHK1i resistance in EC and lays the groundwork for future mechanistic and translational investigations.

## 2. Materials and Methods

### 2.1. Drug Preparation

ATRi camonsertib and CHK1i prexasertib were obtained from the Development Therapeutics Program at National Cancer Institute (Frederick, MD, USA). All drugs were prepared as separate 10 mM stock solutions in dimethyl sulfoxide (DMSO; #S-002-M, MilliporeSigma, Burlington, MA, USA) and stored in aliquots at −80 °C until use.

### 2.2. Cell Lines and Culture Conditions

EC cell lines were selected to represent the major molecular subtypes defined by TCGA. MSI-h models included AN3CA (#HTB-111, ATCC, Manassas, VA, USA), HEC1A (#HTB-112, ATCC), MFE296 (#98031101, MilliporeSigma), and Ishikawa (#99040201, MilliporeSigma). CNH-like cell lines included KLE (#CRL-1622, ATCC), MFE280 (#98050131, MilliporeSigma), and ARK1/ARK2 (gifts from Dr. Alessandro D. Santin, Yale University School of Medicine, New Haven, CT, USA).

Prexasertib-resistant (PrexR; HEC1A-PrexR1, HEC1A-PrexR2, ARK2-PrexR1, ARK2-PrexR2) and camonsertib-resistant (CamR; HEC1A-CamR1, HEC1A-CamR2, ARK2-CamR1, ARK2-CamR2) lines were generated in-house by culturing parental HEC1A and ARK2 cells with increasing concentrations of prexasertib or camonsertib over 3–6 months. All cell lines described above were cultured in RPMI-1640 medium (#11875119, Life Technologies, Frederick, MD, USA), supplemented with 10% fetal bovine serum, 0.01 mg/mL insulin (#I0516, MilliporeSigma), and 1% penicillin-streptomycin at 37 °C in a humidified atmosphere with 5% CO_2_. Resistant cell lines were maintained under continuous selective pressure in the medium containing 1 μM of prexasertib or camonsertib.

All cultures were routinely tested for Mycoplasma contamination using the MycoAlert Detection Kit (#LT-07-318, Lonza, Portsmouth, NH, USA) and confirmed negative before use in experiments.

### 2.3. Cell Growth Assay

2000 cells/well were seeded in 96-well plates and treated with drugs or 0.01% DMSO as control after seeding. After being treated with drugs for 72 h, the cell viability was assessed by the XTT assay (#X6493, Thermo Fisher Scientific, Rockville, MD, USA) according to the manufacturer’s instructions, and the absorbances were measured by Synergy™ HTX Multi-Mode Microplate Reader with Gen5™ software (v3.04) (BioTek Instruments, Winooski, VT, USA). IC_50_ values were calculated using GraphPad Prism v10 (GraphPad Software, Inc., La Jolla, CA, USA).

Cell proliferation was assessed using the IncuCyte S5 Live-Cell Analysis System (Sartorius, Göttingen, Germany). 2000 cells/well were seeded in 96-well plates and allowed to adhere overnight before drug treatment in complete growth medium. Phase-contrast images were acquired every 6 h for 72 h using a 10× objective. Cell confluence was quantified using IncuCyte (v2025C) integrated analysis software and normalized to the initial time point.

### 2.4. RNA Sequencing Analysis

Resistant cells were cultured in drug-free medium for two weeks prior to RNA extraction to minimize residual transcriptional effects of the selection agents. Total RNA was extracted using the RNeasy Mini Kit (#74104, Qiagen, Frederick, MD, USA) with on-column DNase treatment. Libraries were prepared using the Illumina Stranded Total RNA Prep with Ribo-Zero Plus kit (Illumina, San Diego, CA, USA) and sequenced on a NovaSeq X Plus 10B platform to generate 150 bp paired-end reads (~50 million reads per sample). Reads were quality-trimmed using Cutadapt v3.4 and aligned to the hg38 reference genome using STAR v2.7.9a with GENCODE v38 annotations. Gene expression quantification was performed using STAR/RSEM v1.3.3, followed by quartile normalization and log2 transformation.

Gene set enrichment analysis (GSEA) was conducted using GSEA v4.2.3 with the Hallmark gene set collection. Analyses used gene-set permutation (1000 permutations), and pathways with |normalized enrichment score (NES)| > 1.5, nominal *p* values < 0.05 and false discovery rate (FDR) < 0.25 were considered significant. Differential expression analysis was performed using DESeq2 v1.32.0, with significantly differentially expressed genes (DEGs) defined as those with |log2 fold change| > 1 and Benjamini–Hochberg adjusted *p*-value (padj) < 0.05. Functional enrichment of DEGs was assessed using STRING v12.0 (https://string-db.org/) with Gene Ontology (GO) Biological Process terms. Venn diagrams were generated in R (v4.5.1) using the ggVennDiagram package (version 1.5.7).

For heatmap visualization, gene-level RSEM counts were collapsed to unique gene symbols by averaging transcript isoforms, log_2_(x + 1)—transformed and standardized to row-wise Z-scores. Z-score matrices for selected resistance-associated genes were plotted in R (v4.5.1) using the pheatmap package (v1.0.13).

### 2.5. TCGA Survival Analysis

Publicly available endometrial cancer datasets were analyzed using cBioPortal (TCGA Uterine Corpus Endometrial Carcinoma, PanCancer Atlas, https://www.cbioportal.org/study/summary?id=ucec_tcga_pan_can_atlas_2018, accessed on 10 January 2026). mRNA expression levels of *EDN1*, *THBS1*, and *TENM2* were obtained as z-scores relative to diploid samples (RNA Seq V2 RSEM). Patients were stratified into high and low groups using cBioPortal default mRNA expression z-score grouping (relative to diploid samples), and co-expression analyses compared patients with concurrent high *EDN1* and *THBS1* expression to those with low expression of both genes. Disease-specific survival was evaluated using Kaplan–Meier analysis, and statistical significance was assessed by log-rank testing.

### 2.6. Statistical Analysis

All experiments were performed in at least triplicate. XTT assay data are reported as mean ± standard deviation. Correlation between ATRi and CHK1i IC_50_ values was assessed using two-sided Pearson correlation. For RNA sequencing analyses, DEGs were defined by Benjamini–Hochberg padj < 0.05. For GSEA, pathways with |NES| > 1.5, nominal *p* < 0.05, and FDR < 0.25 were considered significant.

## 3. Results

### 3.1. EC Cell Lines Display Correlated Sensitivity to ATR and CHK1 Inhibition

To establish a basis for modeling acquired resistance, we first assessed intrinsic sensitivity to ATRi camonsertib and CHK1i prexasertib across a diverse panel of MSI-h and CNH EC cell lines with different genetic background (Table 1).

**Figure 1:**
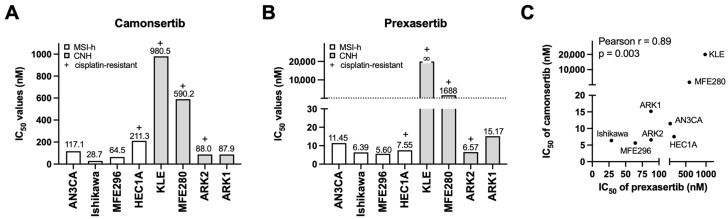
Establishment of the ATRi- and CHK1i-resistant EC cell lines. (**A**,**B**) Cell growth was measured by XTT assays. A panel of EC cell lines were treated with the indicated concentrations of ATRi camonsertib (**A**) and CHK1i prexasertib (**B**) for 3 days and subjected to XTT assays (*n* = 3). IC_50_ values of camonsertib and prexasertib are shown. (**C**) Correlation between IC_50_ of camonsertib and prexasertib in tested EC cell lines.

Sensitivity to ATRi varied by approximately 34-fold (Figure 1A), whereas CHK1i showed a >3500-fold dynamic range (Figure 1B). Despite these differences in magnitude, IC_50_ values for the two inhibitors were strongly correlated (Pearson r = 0.89, *p* = 0.003; Figure 1C), suggesting shared dependence on the ATR-CHK1 pathway.

Based on these profiles, HEC1A (MSI-h) and ARK2 (CNH) were selected as parental models for generating resistant derivatives because both showed sensitivity to ATRi and CHK1i, exhibit platinum resistance, and represent clinically relevant high-risk EC subtypes.

### 3.2. Resistant Lines Exhibit High-Level Resistance and Cross-Resistance Across the ATR-CHK1 Axis

**Figure 2:**
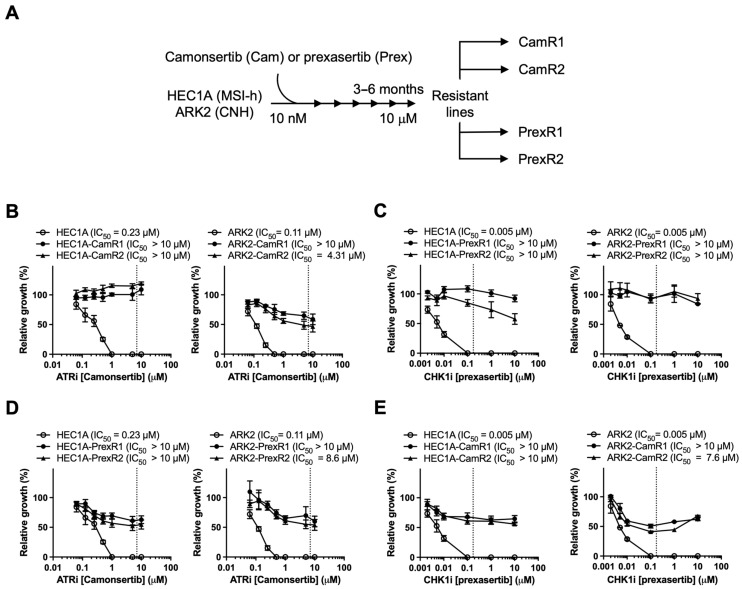
Establishment of the ATRi- and CHK1i-resistant EC cell lines. (**A**) Schematic protocol for generating PrexR and CamR derivatives from parental HEC1A (MSI-h) and ARK2 (CNH) cells is shown. (**C**–**E**) Cells were treated with camonsertib (**B**,**D**) or prexasertib (**C**,**E**) at indicated doses for 3 days and subjected to XTT assays (*n* = 4). Clinically attainable concentrations for prexasertib (0.174 μM) and camonsertib (7 μM) are denoted by the dotted line on each graph.

To model acquired resistance, HEC1A and ARK2 cells were exposed to stepwise increases in camonsertib or prexasertib over 3–6 months, generating two independent ATRi-resistant (CamR1, CamR2) and CHK1i-resistant (PrexR1, PrexR2) clones from each lineage (Figure 2A). XTT assays confirmed substantial resistance: CHK1i-resistant clones demonstrated >2000-fold increases in prexasertib IC_50_ (Figure 2B), and ATRi-resistant lines showed 39–44-fold increases in camonsertib IC_50_ relative to parental cells (Figure 2C). For both inhibitors, resistant IC_50_ values exceeded clinically achievable concentrations.

To assess phenotypic changes associated with resistance, we examined short-term proliferation, morphology, and resistance durability under drug-free conditions. Parental and resistant lines showed comparable baseline growth kinetics and no obvious morphological differences (Figure S1A,B), and resistant clones maintained high-level resistance after 8 weeks of drug withdrawal (Figure S1C), indicating a durable adaptive resistance state.

Cross-resistance analysis showed that PrexR clones were also resistant to ATRi (Figure 2D), and CamR clones displayed comparable resistance to CHK1i (Figure 2E). These findings suggest that prolonged exposure to either inhibitor is associated with reduced responsiveness across the ATR-CHK1 pathway rather than inhibitor-specific resistance alone.

### 3.3. Transcriptomic Profiling Reveals Lineage-Associated and Inhibitor-Associated Enrichment Patterns

**Figure 3:**
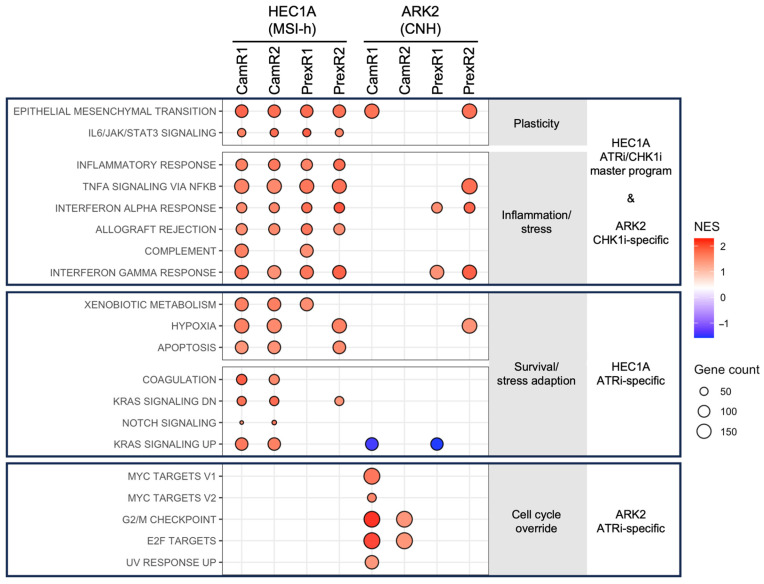
GSEA reveals lineage- and inhibitor-specific transcriptional features in ATRi- and CHK1i-resistant EC cells. GSEA was performed by comparing each resistant clone (CamR or PrexR) with its matched parental HEC1A or ARK2 line. Pathways meeting |NES| > 1.5, nominal *p* < 0.05 and FDR < 0.25 are displayed. Each dot represents a significantly enriched Hallmark pathway in a specific clone; dot size reflects the number of leading-edge genes. Red dots indicate the positive NES and blue dots are negative NES. MSI-h–derived HEC1A-resistant clones (**top**) shared a unified inflammatory-interferon-plasticity transcriptional state, with ATRi-resistant clones (**middle**) showing additional Notch and KRAS pathway enrichment. In the CNH-derived ARK2 lineage (**bottom**), ATRi-resistant clones exhibited strong enrichment of proliferation-associated signatures (G2/M checkpoint, E2F, MYC), whereas CHK1i-resistant clones (**top**) preferentially enriched interferon response pathways. These patterns reveal lineage-defined convergence in MSI-h cells and inhibitor-specific divergence in CNH cells.

To characterize transcriptional features associated with acquired resistance, RNA sequencing was performed on all resistant clones and corresponding parental lines. GSEA of Hallmark pathways revealed clear lineage- and inhibitor-associated enrichment patterns (Figure 3).

Across the MSI-h–derived HEC1A lineage, all resistant clones showed enrichment of pathways related to epithelial–mesenchymal transition (EMT), cytokine signaling (IL6/JAK/STAT3, TNFα/NF-κB), and interferon responses (Figure 3 top block and Figure S2A). These consistent associations suggest a shared transcriptional pattern accompanying resistance in this lineage. ATRi-resistant HEC1A clones also showed additional enrichment of Notch, KRAS, and metabolic gene sets (Figure 3 middle block and Figure S2B), indicating further inhibitor-associated transcriptional differences within this background.

In contrast, transcriptional adaptations diverged in the CNH-derived ARK2 lineage. ATRi-resistant ARK2 clones displayed enrichment of proliferation-associated pathways, including G2/M checkpoint, E2F targets, and MYC targets (Figure 3 bottom block and Figure S2C). CHK1i-resistant ARK2 clones, however, did not show this proliferative enrichment and instead were associated with interferon-related pathways (Figure 3 top block and Figure S2D). Together, these observations indicate that MIS-h HEC1A cells develop a convergent transcriptional state under prolonged ATR or CHK1 inhibition, whereas CNH ARK2 cells exhibit inhibitor-associated differences in pathway enrichment.

### 3.4. Differential Gene Expression Defines Convergent and Inhibitor-Associated Transcriptional Features

To define the gene-level architecture underlying the adaptive states identified by GSEA, we examined differentially upregulated genes in each resistant clone relative to its parental line and assessed shared and inhibitor-specific signatures using Venn analysis and STRING functional enrichment.

#### 3.4.1. MSI-h Lineage: Shared Resistance Module with Additional ATRi-Associated Remodeling

**Figure 4:**
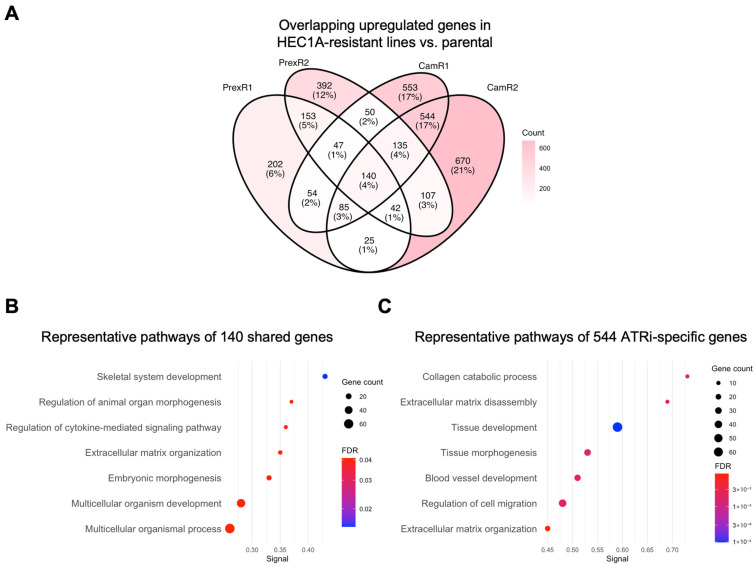
Gene-level analysis of shared and inhibitor-specific upregulated genes in MSI-h HEC1A-derived ATRi- and CHK1i-resistant clones. (**A**) Four-way Venn diagram showing the overlap of upregulated DEGs (|log_2_FC| > 1; padj < 0.05) in HEC1A-CamR1 (Supplementary Table S3), -CamR2 (Supplementary Table S4), -PrexR1 (Supplementary Table S5), -PrexR2 (Supplementary Table S6) compared with parental HEC1A. (**B**) Representative enriched pathways enriched among the 140 shared genes (Supplementary Table S7), highlighting ECM organization, cytokine-mediated signaling, and multicellular development (Supplementary Table S8) in all HEC1A-derived resistant clones. (**C**) Representative enriched pathways for the 544 ATRi-specific genes (Supplementary Table S7), including ECM disassembly, collagen catabolism, and tissue morphogenesis (Supplementary Table S9) in ATRi-resistant HEC1A clones. Dot size indicates gene count; color reflects FDR significance.

Differential expression analysis identified 140 genes consistently upregulated across all HEC1A-derived resistant clones. STRING analysis indicated that these shared genes were associated with extracellular matrix (ECM) organization, cytokine signaling, and developmental or morphogenetic processes (Figure 4A,B), consistent with the pathway-level enrichment patterns (Figure 3, top block).

ATRi-resistant HEC1A clones showed 544 uniquely upregulated genes. These genes were associated with ECM disassembly, collagen catabolism, angiogenesis, and tissue morphogenesis (Figure 4C). These associations suggest that ATRi resistance in HEC1A cells is accompanied by additional transcriptional remodeling beyond the shared resistance module.

CHK1i-resistant HEC1A clones also contained sets of uniquely upregulated genes; however, these gene sets did not yield significant enrichment under the thresholds used for GO enrichment analyses. This may reflect smaller gene set size, greater heterogeneity, or involvement of pathways not well represented in the current annotation databases. Overall, HEC1A-derived resistant clones demonstrated a shared resistance-associated transcriptional pattern, with ATRi resistance showing additional distinct associations.

#### 3.4.2. CNH Lineage: Inhibitor-Associated Transcriptional Differences

**Figure 5:**
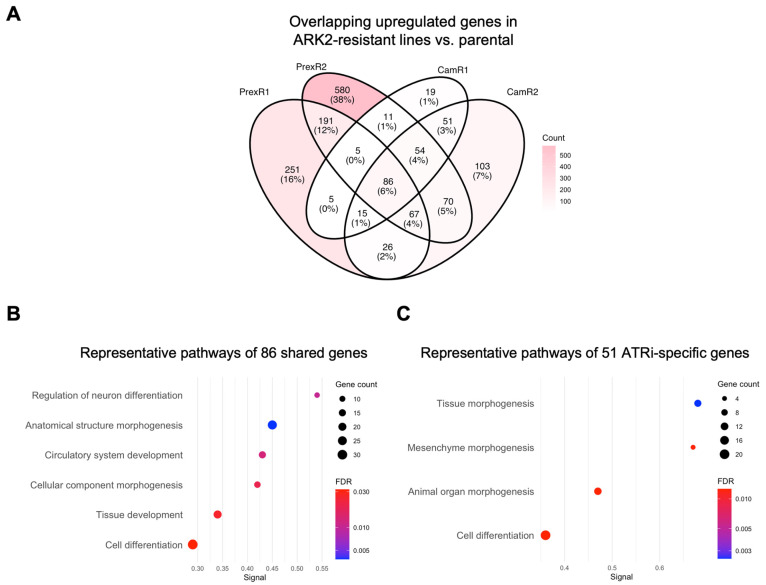
Gene-level analysis reveals shared and inhibitor-specific adaptive modules in CNH ARK2-derived ATRi- or CHK1i-resistant clones. (**A**) Four-way Venn diagram showing the overlap of upregulated DEGs (|log_2_FC| > 1; padj < 0.05) in ARK2-CamR1 (Supplementary Table S10), -CamR2 (Supplementary Table S11), -PrexR1 (Supplementary Table S12), -PrexR2 (Supplementary Table S13) compared with parental ARK2. (**B**) Representative enriched pathways of shared 86 DEGs (Supplementary Table S14) defines a proliferative-morphogenetic core pathways (Supplementary Table S15) in all ARK2-derived-resistant clones. (**C**) Representative enriched pathways of 51 CamR-only upregulated DEGs (Supplementary Table S14) demonstrates a transcription factor-driven developmental remodeling module (Supplementary Table S16) in ATRi-resistant ARK2 clones. Dot size reflects gene count; dot color indicates FDR.

Across all ARK2-derived resistant clones, 86 genes were consistently upregulated relative to parental ARK2 cells. STRING analysis associated these shared genes with morphogenetic and developmental processes, including anatomical structure formation and vascular development (Figure 5A,B). These features may reflect lineage-intrinsic transcriptional characteristics that persist during resistance acquisition.

Inhibitor-associated differences were also observed. ATRi-resistant ARK2 clones showed 51 uniquely upregulated genes that were associated with developmental and morphogenetic processes, including mesenchyme morphogenesis and organ development (Figure 5C). CHK1i-specific DEGs did not show significant pathway-level enrichment, consistent with the more limited gene-level convergence observed under CHK1i in this background.

Together, CNH ARK2-derived resistant clines displayed inhibitor-associated transcriptional differences, with ATRi resistance associated with broader developmental gene expression changes and CHK1i resistance associated primarily with interferon-related pathway enrichment.

#### 3.4.3. Consistency Analysis Identifies a Small Set of Genes Altered Across All Resistant Clones

**Figure 6:**
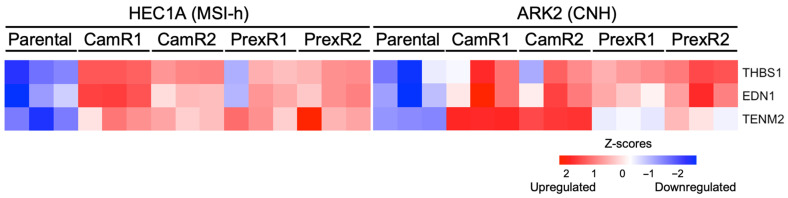
Shared pan-resistant genes across all CHK1i- and ATRi-resistant MSI-h and CNH clones. Heatmap showing *TENM2*, *THBS1*, and *EDN1* genes consistently upregulated across all resistant clones compared to parental HEC1A and ARK2 cell lines.

To identify transcriptomic features shared across ATRi and CHK1i resistance, we analyzed genes that were consistently upregulated in all eight resistant clones. Only three genes, encoding teneurin-2 (*TENM2*), thrombospondin-1 (*THBS1*), and endothelin-1 (*EDN1*), were uniformly increased across both lineages and inhibitor types. These genes define a minimal lineage-independent resistance signature linked to ECM remodeling (TENM2, THBS1 [25,26]) and pro-survival or proliferative signaling (EDN1 [27,28]). Collectively, this signature suggests that resistant cells may adopt an adhesive and survival-oriented transcriptional state that supports continued proliferation under chronic replication stress (Figure 6).

#### 3.4.4. Association of Resistance-Associated Genes with Disease-Specific Survival

To explore the potential clinical relevance of the resistance-associated genes identified in our in vitro findings, we analyzed the TCGA EC cohort. Notably, high expression of *EDN1* and *THBS1*, either individually or in combination, was significantly associated with shorter disease-specific survival (Figure S3). These findings establish a clinical association between components of the in vitro-defined resistance signature and adverse patient outcomes, supporting the relevance of these transcriptional features to aggressive disease biology.

## 4. Discussion

Cell cycle checkpoint inhibition is a promising therapeutic strategy for genomically unstable EC, particularly in MSI-h and CNH tumors [29,30]. While ATRi camonsertib and CHK1i prexasertib have demonstrated clinical activity in subsets of patients with advanced ovarian cancer or EC, responses are often transient [16,17,18,20], highlighting the need to understand how acquired resistance emerges. Notably, our analysis suggests that acquired resistance to ATRi or CHK1i arises independently of baseline cisplatin sensitivity. This observation is consistent with clinical reports showing activity of ATR-CHK1 pathway inhibitors in heavily pretreated, platinum-resistant patients [16,17,18,20], indicating that the selective pressures underlying resistance to cell cycle checkpoint inhibition are distinct from those driving platinum resistance. Our study systematically examines how acquired resistance to ATRi and CHK1i evolves across different EC lineages.

Within this framework, a key finding is that MSI-h HEC1A-derived–resistant clones converge on a similar adaptive transcriptional state under both ATR and CHK1 inhibition. This shared state integrates inflammatory, interferon, EMT, and ECM-remodeling pathways, features associated with cellular plasticity and therapeutic resistance across solid tumors [31,32]. Given the intrinsically inflammatory signaling environment of MSI-h EC [9], these tumors may be predisposed to adopt such adaptive programs under cell cycle checkpoint inhibition. While ATRi resistance introduces additional developmental and KRAS/Notch-related remodeling, the overarching resistance feature remains conserved within this lineage.

In contrast to the convergent behavior observed in MSI-h HEC1A models, CNH ARK2-derived–resistant clones exhibit inhibitor-specific transcriptional adaptations. ATRi resistance is characterized by enrichment of proliferation-linked programs, whereas CHK1i resistance preferentially activates interferon-related pathways. Such divergence has not been widely reported in other *TP53*-mutant cancers treated with cell cycle checkpoint inhibitors, where CHK1i resistance has often been attributed to alterations in cell cycle checkpoints and CDK regulation [23], and ATRi resistance has been linked to specific modulators such as CDC25A or nonsense-mediated decay factors [21,22]. Our findings suggest that ATR and CHK1 inhibition impose distinct selective pressures in CNH EC despite a shared replication stress background. Notably, this divergence persists even in the context of cross-resistance, indicating that similar resistance phenotypes can arise through multiple, non-overlapping evolutionary trajectories.

Despite these lineage- and inhibitor-specific differences, only three genes, *THBS1*, *EDN1* and *TENM2*, were consistently upregulated across all resistant models. THBS1 and TENM2 have been implicated in ECM organization and invasion [25,26], while EDN1 is linked to proliferative and treatment-resistant phenotypes in multiple malignancies [27,28]. Although this three-gene set has not previously been associated with resistance to cell cycle checkpoint inhibitors, its consistent induction across EC lineages and inhibitor classes suggests the existence of a minimal, lineage-independent pan-resistance module that warrants further investigation.

From a hypothesis-generating perspective, our findings also suggest potential directions for subtype-specific therapeutic strategies. In MSI-h EC, adaptive states enriched for inflammatory and EMT-related programs raise the possibility that targeting JAK/STAT or NF-κB pathway inhibition may enhance the durability of cell cycle checkpoint inhibition [33]. In CNH EC, ATRi-associated proliferation and MYC/E2F enrichment point to CDK- or MYC-directed agents as rational combinational partners [34,35], whereas the interferon-associated signature in CHK1i resistance raises questions about whether innate immune pathways contribute directly to adaptation. Collectively, these lineage-associated transcriptional signatures provide a framework for biomarker development. In particular, EDN1 and THBS1 may represent candidate markers of emerging resistance, potentially amenable to longitudinal monitoring through tumor biopsies or circulating RNA-based assays.

Several considerations should be acknowledged when interpreting these findings. First, this study relied on in vitro isogenic models, which capture cell-intrinsic transcriptional adaptations but do not account for tumor microenvironmental influences. In addition, lineage-associated observations were derived from representative MSI-h HEC1A and CNH ARK2 models and may vary across additional EC backgrounds. Importantly, these descriptive transcriptomic data require future proteomic validation to bridge the functional gap between mRNA signatures and protein expression within a multi-omics framework [36]. Our findings should also be considered hypothesis-generating and the identified resistance-associated drivers, such as EDN1 and THBS1 will require validation using CRISPR-based or pharmacologic approaches. While the current lack of public transcriptomic datasets from ATRi- or CHK1i-treated EC patients precludes direct clinical validation, our findings establish a foundational framework that can be tested as these targeted agents continue to advance in clinical development.

## 5. Conclusions

In summary, our study identifies lineage- and inhibitor-specific transcriptomic characteristics that accompany acquired ATRi and CHK1i resistance in EC. MSI-h HEC1A-derived–resistant cells exhibit a convergent inflammatory and EMT-associated pattern, whereas CNH ARK2-derived–resistant EC clones show inhibitor-specific adaptive profiles. These findings provide a molecular framework for understanding resistance-associated transcriptional states and may guide future efforts to define biomarkers or lineage-informed treatment approaches for advanced EC.

## Figures and Tables

**Table 1:** Baseline characteristics of EC cell lines used in this study.

				Mutation Status					
Cell Line	Histology	Subtype	Cisplatin	*TP53*	*MSH6*	*PMS2*	*PIK3CA*	*PIK3R1*	*PTEN*
AN3CA	Endometrioid	MSI-h	Sensitive	R213Q	F1088SfsTer2	wildtype	wildtype	R557_K561>Q	R130Q
Ishikawa	Endometrioid	MSI-h	Sensitive	M246V	I1183T, F1088SfsTer2	Q604R	wildtype	L570P	V317fs
HEC1A	Endometrioid	MSI-h	Resistant	R248G	F1088SfsTer2	R802Ter	G1049R	wildtype	wildtype
MFE296	Endometrioid	MSI-h	Sensitive	Y220C	A1055V	wildtype	P539R	wildtype	R130Q
KLE	Endometrioid	CNH	Resistant	R175H	wildtype	wildtype	wildtype	wildtype	wildtype
MFE280	Endometrioid	CNH	Resistant	X307_splice	wildtype	wildtype	H1047Y	wildtype	wildtype
ARK1	Serous	CNH	Sensitive	R248W	wildtype	wildtype	E542K	wildtype	wildtype
ARK2	Serous	CNH	Resistant	Q165Ter	wildtype	wildtype	wildtype	wildtype	wildtype

Notes: Mutation status was obtained from the Cancer Cell Line Encyclopedia via cBioPortal (https://www.cbioportal.org/) and Cellosaurus (https://www.cellosaurus.org/). *TP53* status was validated by in-house next-generation sequencing. Subtypes were defined by their molecular background based on TCGA subgroups [9]. Ter indicates termination.

## Data Availability

The original contributions presented in this study are included in the article/Supplementary Materials. Further inquiries can be directed to the corresponding author(s).

## Supplementary Materials

The following supporting information can be downloaded at: https://www.mdpi.com/article/10.3390/biom16010169/s1, Table S1: GSEA of baseline ATRi- or CHK1i-resistant clones versus parental HEC1A cells. Table S2: GSEA of baseline ATRi- or CHK1i-resistant clones versus parental ARK2 cells. Table S3: DEGs of baseline HEC1A-CamR1 versus parental HEC1A cells. Table S4: DEGs of baseline HEC1A-CamR2 versus parental HEC1A cells. Table S5: DEGs of baseline HEC1A-PrexR1 versus parental HEC1A cells. Table S6: DEGs of baseline HEC1A-PrexR2 versus parental HEC1A cells. Table S7: Overlapping upregulated genes in HEC1A-CamR and HEC1A-PrexR clones versus parental HEC1A cells. Table S8: STRING pathway analysis of 140 overlapping upregulated genes in HEC1A-CamR and HEC1A-PrexR clones versus parental HEC1A cells. Table S9: STRING pathway analysis of 544 unique upregulated genes in HEC1A-CamR clones versus parental HEC1A cells. Table S10: DEGs of baseline ARK2-CamR1 versus parental ARK2 cells. Table S11: DEGs of baseline ARK2-CamR2 versus parental ARK2 cells. Table S12: DEGs of baseline ARK2-PrexR1 versus parental ARK2 cells. Table S13: DEGs of baseline ARK2-PrexR2 versus parental ARK2 cells. Table S14: Overlapping upregulated genes in ARK2-CamR and ARK2-PrexR clones versus parental ARK2 cells. Table S15: STRING pathway analysis of 86 overlapping upregulated genes in ARK2-CamR and ARK2-PrexR clones versus parental ARK2 cells. Table S16: STRING pathway analysis of 51 unique upregulated genes in ARK2-CamR clones versus parental ARK2 cells. Figure S1. Phenotypic characterization and durability of ATRi- and CHK1i-resistant EC cells. Figure S2. Classical GSEA enrichment plots for selected Hallmark pathways in ATRi- and CHK1i-resistant EC cells. Figure S3. Association of resistance-associated genes with disease-specific survival in TCGA endometrial cancer.

## Abbreviations

The following abbreviations are used in this manuscript:

ATR	Ataxia telangiectasia and Rad3-related kinase
ATRi	ATR inhibitor
CamR	Camonsertib-resistant
CHK1	Cell cycle checkpoint kinase 1
CHK1i	Cell cycle checkpoint kinase 1 inhibitor
CNH	Copy-number-high
CRISPR	Clustered regularly interspaced short palindromic repeats
DEG	Differentially expressed gene
EC	Endometrial cancer
ECM	Extracellular matrix
EDN1	Endothelin-1
EMT	Epithelial–mesenchymal transition
FDR	False discovery rate
GDF15	Growth/differentiation factor 15
GO	Gene ontology
GSEA	Gene set enrichment analysis
IL6	Interleukin 6
JAK	Janus kinases
MSI-h	Microsatellite instability-high
NES	Normalized enrichment score
NF-κB	Nuclear factor kappa-light-chain-enhancer of activated B cells
padj	Benjamini–Hochberg adjusted *p*-value
PrexR	Prexasertib-resistant
STAT3	Signal transducer and activator of tanscription 3
TENM2	Teneurin-2
THBS1	Thrombospondin-1
TNFα	Tumor necrosis factor-alpha
