# Supplementary material for: Transcriptional Profiling Reveals Lineage-Specific Characteristics in ATR/CHK1 Inhibitor-Resistant Endometrial Cancer
Source: Biomolecules. 2026 Jan 20;16(1):169. doi: 10.3390/biom16010169 (PMC12839321; doi:10.3390/biom16010169)
Supplement: Supplementary file 1 [file biomolecules-16-00169-s001.zip › Supplementary Figures.pdf]

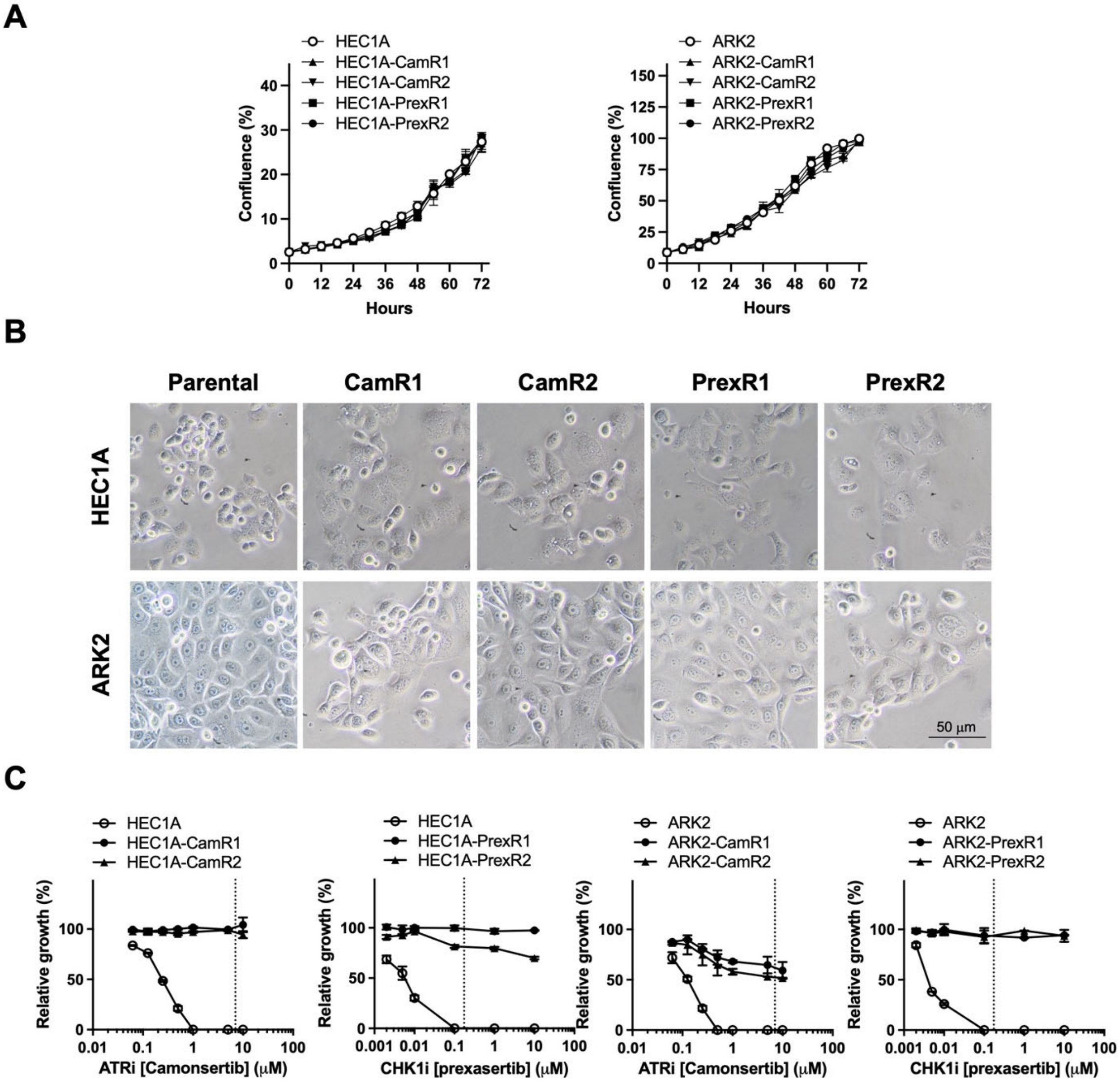

Figure S1. Phenotypic characterization and durability of ATRi- and CHK1i-resistant EC cells. (**A**) Real-time IncuCyte confluence monitoring over 72 hours under drug-free conditions shows comparable proliferation kinetics between parental and resistant lines. (**B**) Representative phase-contrast images demonstrate no overt morphological differences between parental and resistant cells. (**C**) Resistant cells were cultured in drug-free media for 8 weeks prior to reassessment of drug sensitivity and maintained resistance to ATRi or CHK1i, indicating durable resistance after prolonged drug withdrawal. Scale bar, 50 μm.

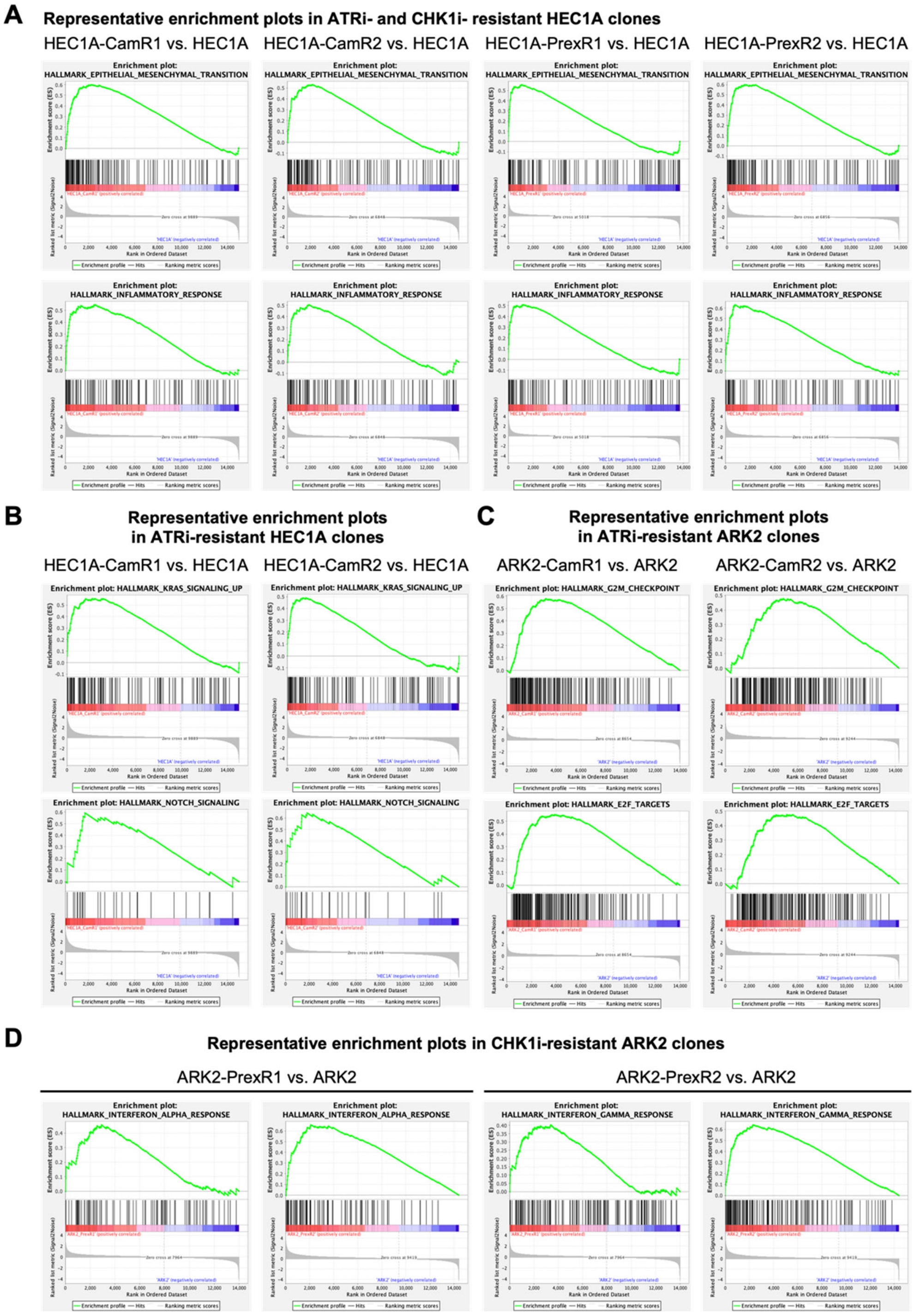

Figure S2. Classical GSEA enrichment plots for selected Hallmark pathways in ATRi- and CHK1i-resistant EC cells. (**A**–**D**) Classical GSEA enrichment plots showing running enrichment scores, gene set hit distributions, and ranked gene-level statistics for selected Hallmark pathways. Panels depict representative major pathways enriched in all HEC1A-derived resistant clones (**A**), ATRi-resistant HEC1A (**B**), ATRi-resistant ARK2-resistant clones (**C**) and CHK1i-resistant ARK2-resistant cell lines (**D**).

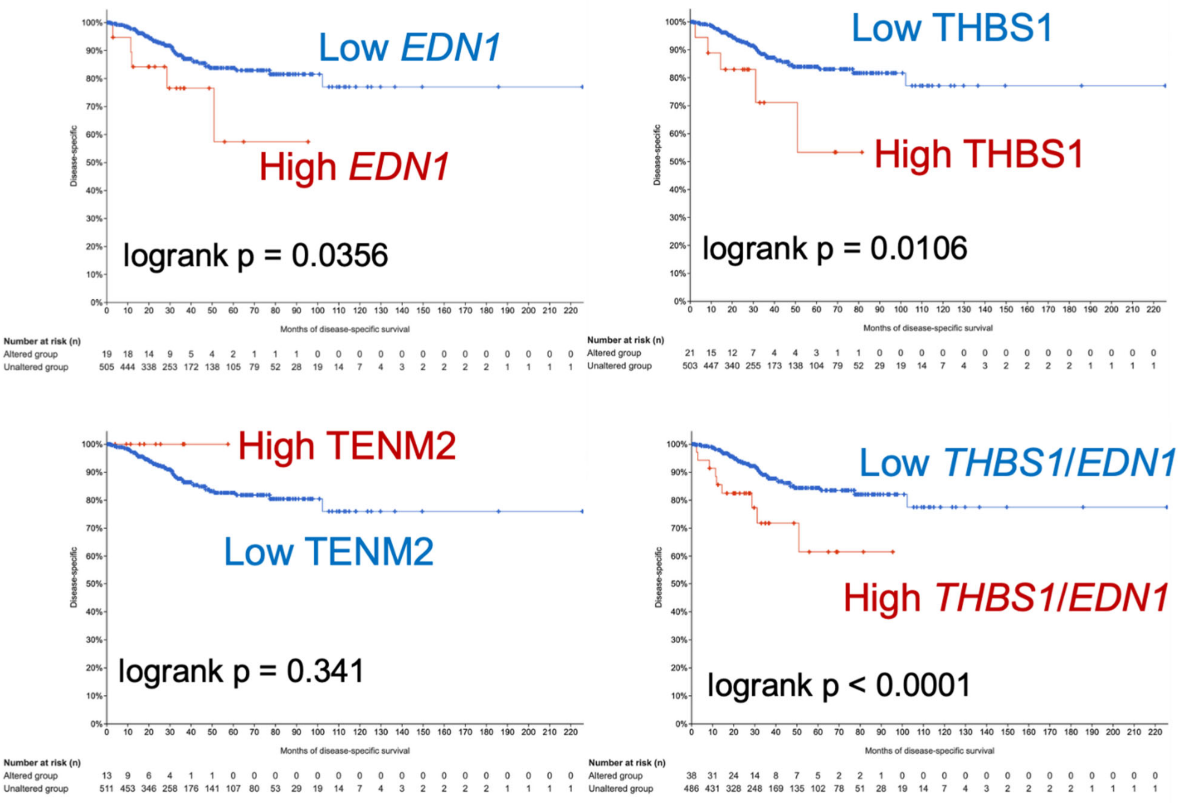

Figure S3. Association of resistance-associated genes with disease-specific survival in TCGA endometrial cancer. Kaplan-Meier analyses of disease-specific survival in endometrial cancer patients (TCGA Uterine Corpus Endometrial Carcinoma dataset, PanCancer Atlas, https://www.cbioportal.org/) stratified by mRNA expression levels of *EDN1*, *THBS1*, and *TENM2*. Higher expression of *EDN1* or *THBS1* was associated with worse disease-specific survival, whereas *TENM2* alone was not prognostic. Co-expression analysis demonstrated that patients with concurrent high *EDN1* and *THBS1* expression exhibited significantly poorer disease-specific survival compared with those with low expression of both genes. Log-rank p values are indicated. These analyses assess prognostic associations and do not reflect response to ATR or CHK1 inhibition.
